# Outcome and Midterm Survival after Heart Transplantation Is Independent from Donor Length of Stay in the Intensive Care Unit

**DOI:** 10.3390/life12071053

**Published:** 2022-07-14

**Authors:** Daniel Oehler, Charlotte Böttger, Moritz Benjamin Immohr, Raphael Romano Bruno, Jafer Haschemi, Daniel Scheiber, Patrick Horn, Hug Aubin, Igor Tudorache, Ralf Westenfeld, Payam Akhyari, Malte Kelm, Artur Lichtenberg, Udo Boeken

**Affiliations:** 1 Division of Cardiology, Pulmonology and Vascular Medicine Medical Faculty, Heinrich-Heine University, 40225 Düsseldorf, Germany; raphael.bruno@med.uni-duesseldorf.de (R.R.B.); jafer.haschemi@med.uni-duesseldorf.de (J.H.); daniel.scheiber@med.uni-duesseldorf.de (D.S.); patrick.horn@med.uni-duesseldorf.de (P.H.); ralf.westenfeld@med.uni-duesseldorf.de (R.W.); malte.kelm@med.uni-duesseldorf.de (M.K.); 2 Department of Diagnostic and Interventional Radiology, Medical Faculty, Heinrich-Heine University, 40225 Düsseldorf, Germany; charlotte.boettger@med.uni-duesseldorf.de; 3 Department of Cardiac Surgery, Medical Faculty, Heinrich-Heine University, 40225 Düsseldorf, Germany; moritz.immohr@med.uni-duesseldorf.de (M.B.I.); hug.aubin@med.uni-duesseldorf.de (H.A.); igor.tudorache@med.uni-duesseldorf.de (I.T.); payam.akhyari@med.uni-duesseldorf.de (P.A.); artur.lichtenberg@med.uni-duesseldorf.de (A.L.)

**Keywords:** donor stay in ICU, midterm survival, heart transplantation

## Abstract

Prolonged treatment of organ donors in the intensive care unit (ICU) may be associated with complications influencing the outcome after heart transplantation (HTx). We therefore aim to explore the potential impact of the donor length of stay (LOS) in the ICU on outcomes in our cohort. We included all patients undergoing HTx in our center between September 2010 and April 2022 (*n* = 241). Recipients were divided around the median into three groups regarding their donor LOS in the ICU: 0 to 3 days (≤50th percentile, *n* = 92), 4 to 7 days (50th–75th percentile, *n* = 80), and ≥8 days (≥75th percentile, *n* = 69). Donor LOS in the ICU ranged between 0 and 155 days (median 4, IQR 3–8 days). No association between the LOS in the ICU and survival after HTx was observed (AUC for overall survival 0.514). Neither the Kaplan–Meier survival analysis up to 5 years after HTx (Log-Rank *p* = 0.789) nor group comparisons showed significant differences. Baseline recipient characteristics were comparable between the groups, while the donor baselines differed in some parameters, such as less cardiopulmonary resuscitation prior to HTx in those with a prolonged LOS. However, regarding the recipients’ peri- and postoperative parameters, the groups did not differ in all of the assessed parameters. Thus, in this retrospective analysis, although the donors differed in baseline parameters, the donor LOS in the ICU was not associated with altered recipient survival or outcome after HTx.

## 1. Introduction

The effect of donor parameters on outcome and survival after heart transplantation is crucial and is currently one of the most essential topics of organ acceptance [1,2,3,4,5,6,7]. A potential donor risk factor for organ quality is the donor length of stay (LOS) in the ICU. In general, out of the context of solid organ transplantation, the LOS in the ICU is associated with reduced long-term survival in elderly critically ill patients [8]. Additionally, independent of age, and especially in the initial period of 5–10 days, the probability of future survival does decrease with increasing LOS in unselected adult patients admitted to ICUs [9]. Regarding heart transplantation, this raises the question of the potential impact of donors’ LOS in the ICU on the recipient’s outcome and survival. The definition of a short or long stay in the ICU seems crucial in this manner. However, the reported durations differ widely in the literature, ranging from >3 to >10 days [8,10,11]. Additionally, only a few published data explicitly focus on the influence of donor LOS in the ICU or IMC and the respective outcome of solid organ transplant recipients. Furthermore, information regarding the influence of donor LOS in the ICU on the recipient’s outcome and survival, specifically in an adult heart transplant cohort, is limited. Other parameters, such as the duration of heart ischemic time and impact of high-dose inotropic support in donors may play an additional role here [12,13]. Regarding other solid organ transplantation, data from adult liver transplant recipients present a heterogeneous pattern, as some studies reported an impact of donor LOS on survival [14,15,16], while others reported no significant effect [17,18,19]. Therefore, this study aims to fill this gap by investigating a potential association between the donor length of stay in the ICU and morbidity and midterm survival up to 5 years after heart transplantation.

## 2. Materials and Methods

### 2.1. Ethics

This study conformed to the principles of the Declaration of Helsinki and Good Clinical Practices. All heart transplant recipients participated voluntarily and provided informed consent. The study was approved by our local ethics committee.

### 2.2. Recipients and Study Design

We screened all patients undergoing HTx in our center between September 2010 and April 2022 (*n* = 241), of which all had complete data on the length of donor stay in the ICU according to the corresponding donor report and were therefore included. The cohort was split into groups around the median dividing the recipients into three groups in regard to their donor LOS in the ICU: Group 1, 0 to 3 days (≤50th percentile, *n* = 92); group 2, 4 to 7 days (50th–75th percentile, *n* = 80); and group 3, ≥8 days (≥75th percentile, *n* = 69).

### 2.3. Data Collection

All relevant recipient and donor variables were reviewed and compared between the five groups. Recipient and donor characteristics and recipient survival of up to 5 years, including 30 days and 1 year after transplantation, were collected where applicable.

### 2.4. Statistical Analysis and Figure Making

Association between the donor LOS and survival after HTx was investigated using receiver operating characteristics (ROC) analysis and the Kaplan–Meier survival method. Qualitative (dichotomous) variables were compared by Pearson’s chi-squared test or, when its application conditions were not met, by Fisher’s exact test. If one of the two groups contained a zero value for an event, Yates’ correction was added. Quantitative variables were compared using Student’s *t*-test. The tests were performed bilaterally, and the threshold of significance was set at 0.05. Statistical analysis was performed using GraphPad Prism and IBM SPSS Statistics software (SPSS). Figures were created using GraphPad Prism, Microsoft PowerPoint, and IBM SPSS.

## 3. Results

### 3.1. Recipient Data

Baseline characteristics in recipients were comparable between the groups, including the parameters of size mismatch and comorbidities. Furthermore, no statistically relevant differences could be observed in the laboratory values, including recipient sodium and potassium levels as well as creatinine, bilirubin, and hemoglobin (see Table 1).

### 3.2. Donor Data

The donor length of stay in the ICU ranged between 0 and 155 days (median 4 days, IQR 3–8 days). Donors with the highest duration in the ICU had less cardiopulmonary resuscitation (CPR) prior to organ retrieval, which was significant in comparison to those with a medium LOS (16 vs. 39%, *p* = 0.003).

Intracerebral bleeding (ICB) as a donor cause of death was less frequent in donors with a medium in comparison to a short and prolonged LOS (29% vs. 50% resp. 61%, *p* = 0.005 resp. 0.0001). In those with a prolonged LOS, traumatic brain injury was less likely to be reported as a cause of death and thereby significantly different to those with a medium LOS (13% vs. 26%, *p* = 0.05). Regarding laboratory values, those with a medium duration in the ICU had significantly higher donor sodium levels (153 mmol/L, IQR 145–158 mmol/L) than those with short (147 mmol/L, IQR 142–151 mmol/L, *p* = <0.0001) and long (149 mmol/L, IQR 143–154 mmol/L, *p* = 0.017) stays. Additionally, longer duration in the ICU was associated with decreasing hemoglobin levels, with 11 g/dL in the short and 9 g/dL in the long duration group, reaching significance between the short and prolonged LOS groups (*p* = 0.00011) and 2 vs. 3 (*p* = 0.025).

All other baseline characteristics and laboratory values were comparable, including other parameters such as size mismatch, the left ventricular ejection fraction, and comorbidities as well as donor potassium levels, lactate dehydrogenase, peak white blood cell count, and C-reactive protein (see Table 2).

### 3.3. Perioperative Morbidity

Regarding peri- and postoperative parameters, the groups did not differ in cold or total graft ischemia time, length of postoperative hospital or IMC/ICU stay, duration of mechanical ventilation, duration of surgery, or need for transfusion of packed red blood cells, platelets, or fresh frozen plasma (see Table 3). In all of the included patients, the bicaval technique for heart transplantation was used. Cardioprotection was performed in all patients via cold storage with crystalloid cardioplegia.

With respect to common postoperative morbidities, patients are comparable in the occurrence of severe infection or sepsis, the incidence of acute graft rejection (>1R), the likelihood of kidney failure with hemodialysis post-HTx, postoperative neurological complications, the frequency of re-sternotomy post-HTx, or the need for mechanical life support post-HTx.

### 3.4. Survival

**Figure 1:**
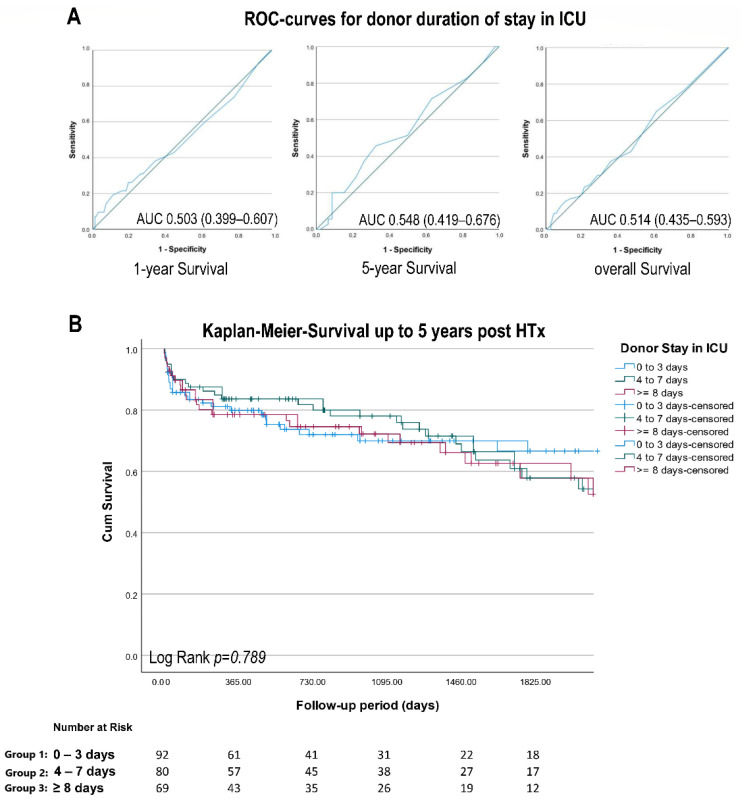
Association of donor length of stay in the ICU and survival. (**A**) Receiver operating characteristics (ROC) analysis for 1-year, 5-year, and overall survival. Area under the curve (AUC) shows no association for either 1-year (AUC 0.503), 5-year (AUC 0.548), or overall survival (AUC 0.514); (Log-Rank *p* = 0.789). (**B**) The Kaplan–Meier survivals for short (0 to 3 days, *n* = 92), medium (4 to 7 days, *n* = 80), and long (≥8 days, *n* = 69) donor length of stay in the ICU prior to organ removal. Data under the graph represent patients at risk at specific time points in all groups. For details see text.

No clear general association between donor stay in the ICU and survival after HTx was observed using receiver operating characteristic (ROC) analysis (see Figure 1A): AUC for 1-year survival, 0.503 (95% CI 0.399–0.607); AUC for 5-year survival, 0.548 (95% CI 0.419–0.676); and AUC for overall survival, 0.514 (0.435–0.593). Thirty-day and one-year survival was also comparable between all three groups (Table 1), and these results were confirmed by the Kaplan–Meier survival analysis (Figure 1B), showing the equality of survival distribution across all groups (Log-Rank χ^2^ 0.229, *p* = 0.79).

## 4. Discussion

Only limited knowledge exists on the association of perioperative morbidity and mortality in heart transplant recipients and the duration of the donor ICU/IMC stay prior to organ removal. We therefore aimed to investigate the influence of different levels of donor LOS in the ICU/IMC on outcomes after heart transplantation, and thus retrospectively analyzed 241 HTx recipients in a 10-year study period.

Here, our main finding is that despite differing in a few donor baseline characteristics, donor duration in the ICU/IMC did not influence midterm survival up to 5 years after heart transplantation.

There is little data explicitly focusing on the influence of donor LOS in the ICU or IMC and the respective outcome of solid organ transplant recipients, especially for heart transplantation. Our study’s median donor LOS in the ICU/IMC was 4 days (IQR 3–8 days). This is in line with the published data from Australia with a mean LOS in the ICU of 3 days [20] and a pediatric liver transplantation cohort with a mean LOS of also 3 days [20]. In a larger cohort study by Smits et al., the majority of all donors had ICU LOS ranging between 1 and 6 days [3]. Additionally, in general, the average ICU LOS independent from the underlying disease is 3.3 days [21], matching the donor LOS in our cohort. However, larger cohort data are unavailable because most authors, even in papers focusing on heart transplant donor parameters, do not report the donor ICU LOS [2,22,23]. Of note, in a recent study by D’Aragon et al. investigating overall donor characteristics in Canada (DONATE cohort study [24]), donors with the neurological determination of death (donation after brain dead; DBD) had shorter LOS in the ICU prior to solid organ donation than those with the circulatory determination of death (DCD, 1.6 vs. 3.8 days). The influence of this shorter period on outcomes, however, is unclear. In our center, due to legal reasons, only donors with DBD can be accepted for transplantation. Therefore, we cannot exclude a potential bias regarding worse outcomes by NDD vs. DCD in general. However, the absolute number of used donors with DCD for heart transplantations in the whole Eurotransplant area was only *n* = 4 in 2019 (Annual Report 2019), and in the mentioned DONATE study, no heart transplantation was performed from DCD donors. Thus, the absolute potential influence of DCD vs. NDD for heart transplantation in our reference cohorts seems to be low.

Concerning baseline characteristics, recipients’ preoperative parameters were comparable between the groups. However, in the present study, donors with the longest duration in the ICU had less frequent CPR (16%) compared to the other groups (all patients and short LOS 29%, medium LOS 39%). This can partly be explained by a combination of higher and earlier mortality under those patients with CPR in the ICU in general [25], leading to a shorter LOS before brain death and therefore a negative selection bias concerning CPR in those with a longer LOS.

In our cohort, donors with a medium LOS died from ICB less frequently, while in donors with prolonged ICU LOS, traumatic brain injury was less frequent. Unfortunately, with the donor reports’ underlying data, we cannot state the reasons for this phenomenon. However, it was recently shown that the donor cause of death could be associated with a reduced recipient survival rate after heart transplantation [4]. Although we did not encounter differences in survival rates between the groups, this could lead to a potential bias in the current study that we cannot exclude.

Regarding donor laboratory values, two parameters differed between the groups. First, those with a medium LOS had slightly higher sodium levels (153 mmol/L vs. 149 mmol/L in all patients and 147 and 149 mmol/L in the other groups). In general, the cut-off values for severity of hypernatremia range from >150 mmol/L [26] to >156 mmol/L [27]. Controversial and limited data exist regarding the impact of this donor sodium dysregulation in patient survival after solid organ transplantation, with cut-off levels for influence on survival ranging between 159 and 170 mmol/L [28,29]. Applying these cut-off levels to our cohort, those with a medium LOS in the ICU would be classified as mildly hypernatremic. According to the cited data, these small changes in sodium levels are most likely without relevance to outcome or survival in our cohort. However, this is obviously limited by the stated shortage of data available on this topic. Additionally, in our cohort, there was a coincidence between mild hypernatremia and a higher frequency of CPR prior to organ removal in the medium LOS group. Although maybe only coincidentally, we cannot exclude that those two parameters are correlated in terms of both pathophysiological electrolyte changes due to the CPR and treatment with medication with high sodium content during CPR, such as sodium bicarbonate.

Increasing donor LOS was associated with lower hemoglobin levels in our cohort, from the shortest to highest LOS ranging from 11 g/dL over 10 g/dL to 9 g/dL. This goes in line with data from Misar et al. in a large pediatric liver transplant cohort [30], as they could observe a decrease in hemoglobin in association with the donor LOS in the ICU. Unfortunately, limited by the information on the donor report, we cannot investigate further explanations; however, it could be speculated that blood transfusion triggers were set at lower levels than usual around the time of brain death. This is supported by the data that show that usually in critically ill patients, a longer LOS in the ICU is associated with a higher rate of transfusion of red blood cells due to decreasing hemoglobin levels [10].

Regarding peri- and postoperative morbidity, the groups did not differ in all parameters assessed in this study, including common postoperative morbidities. Concerning survival, no clear general association between donor stay in the ICU and survival after HTx was observed using receiver operating characteristic (ROC) analysis. Thirty-day and one-year survival was equally distributed between all three groups. Compared to the literature, the 30-day survival in our cohort (ranging from 87–94%) is comparable to that of larger HTx cohorts (89–93% [31,32]). The influence of the donor length of stay in the ICU on the recipient’s outcome and survival after heart transplantation has, to our knowledge, not yet been investigated in an adult heart transplant cohort. Regarding other solid organ transplantations, there is, however, limited and controversial data on adult liver transplant recipients, with some data showing an impact of donor LOS on survival [14,15,16], while others do not [17,18,19]. Additionally, data from the pediatric liver transplant cohort by Misar et al. [30], although limited by a small sample size of *n* = 75 in total, suggest no impact on recipient survival, but longer donor LOS in the ICU was associated with increased biliary complication, one of the major complications after liver transplantation. Although biliary complications do not typically arise for heart transplant recipients, this shows that in other solid organ transplantations, a specific major complication was associated with a prolonged LOS in the ICU.

This study’s major and obvious limitation is that it is composed of a single center and only includes retrospective data. Additionally, this study is technically not able to investigate the possible pathomechanistical (biochemical) reasons behind the reported association. Therefore, future studies with larger cohorts, preferably from the newest era of heart transplantation and potentially with a prospective design and relationship to basic science, are needed to confirm or deny associations of donor length of stay in the ICU on outcome and survival after heart transplantation.

## 5. Conclusions

In this retrospective analysis, although differing in some baseline parameters, the donor length of stay in the ICU was not associated with altered survival or outcome after HTx. Future studies have yet to be performed to confirm these findings on a prospective and larger scale.

## Figures and Tables

**Table 1:** Preoperative recipient parameters. Preoperative recipient data regarding the donor length of stay in the ICU grouped by percentiles around the median (group 1–3). Median values with interquartile range (Q1–Q3) are shown for continuous variables or percentages for discrete variables. Significance was calculated by Pearson’s chi-square test or, when its application conditions were not met, by Fisher’s exact test for qualitative variables and Student’s *t*-test for quantitative variables.

Recipient Variables	All Patients	Gr 1 0–3 Days	Gr 2 4–7 Days	Gr 3 ≥8 Days	*p*		
	*n* = 241	Total *n* = 92	Total *n* = 80	Total *n* = 69	1 vs. 2	1 vs. 3	2 vs. 3
Age (y)	58 (50–62)	59 (51–62)	57 (45–63)	59 (53–63)	0.18	0.47	0.06
Gender (% male)	72.2	73.9	75.0	66.7	>0.99	0.38	0.28
Height (cm)	175 (169–180)	175 (168–182)	175 (170–180)	173 (168–178)	0.66	0.29	0.50
Weight (kg)	78 (68–87)	80 (69–90)	79 (70–87)	74 (65–83)	0.95	0.06	0.07
Body mass index (kg/m^2^)	25 (23–28)	26 (23–29)	26 (23–29)	24 (21–28)	0.82	0.10	0.09
Predicted heart mass ratio (%)	0.98 (0.87–1.10)	0.99 (0.89–1.07)	0.98 (0.84–1.14)	0.97 (0.88–1.12)	0.71	0.47	0.76
Cardiac reoperation (%)	63.1	62.0	65.0	62.3	0.75	>0.99	0.86
High-urgency waiting list (%)	44.8	37.0	48.8	50.7	0.13	0.11	0.87
Ventricular assist device (%)	50.6	50.0	50.0	52.2	>0.99	0.87	0.87
CPR pre HTx (%)	7.3	13.0	10.1	15.9	0.72	0.83	0.27
Diabetes mellitus (%)	22.6	17.6	24.1	27.5	0.34	0.18	0.71
Arterial hypertension (%)	57.1	59.8	60.8	49.3	>0.99	0.20	0.19
ICM (%)	43.5	53.3	37.2	37.7	0.06	0.06	>0.99
Pulmonary hypertension (%)	9.1	12.0	8.9	5.8	0.62	0.27	0.54
PRA pre HTx (% as mean)	2.33 (±13.7)	0.82 (±5.2)	3.8 (±19.3)	2.63 (±13.5)	0.18	0.30	0.68
PRA >10% pre HTx (*n*, %)	7/241 (2.9	1/92 (1.1)	3/80 (3.7)	3/69 (4.3)	0.34	0.31	>0.99
Laboratory values							
Hemoglobin (g/dL)	12 (10–14)	12 (10–14)	12 (10–14)	12 (10–13)	0.85	0.78	0.93
Creatinine (mg/dL)	1.2 (1.0–1.6)	1.2 (1.0–1.6)	1.1 (1.0–1.5)	1.2 (0.9–1.7)	0.47	0.37	0.87
GFR pre HTx (mL/min)	62 (45–82)	63 (47–81)	65 (48–81)	60 (39–85)	0.71	0.53	0.35
Bilirubin (mg/dL)	0.6 (0.4–1.0)	0.6 (0.4–1.0)	0.6 (0.4–1.1)	0.6 (0.4–1.0)	0.37	0.48	0.86
Lactate dehydrogenase (U/L)	254 (213–314)	248 (212–307)	259 (211–328)	258 (224–316)	0.47	0.44	0.14
Sodium (mmol/L)	138 (136–141)	139 (136–141)	138 (136–140)	138 (137–141)	0.37	0.66	0.20
Potassium (mmol/L)	4.3 (3.9–4.6)	4.3 (3.9–4.6)	4.3 (3.9–4.6)	4.2 (3.9–4.6)	0.25	0.25	0.99

**Table 2:** Preoperative donor parameters. Preoperative donor data regarding the donor length of stay in the ICU grouped by percentiles around the median (group 1–3). Median values with interquartile range (Q1–Q3) are shown for continuous variables or percentages for discrete variables. Significance was calculated by Pearson’s chi-square test or, when its application conditions were not met, by Fisher’s exact test for qualitative variables and Student’s *t*-test for quantitative variables. Bold *p*-values represent statistical significance.

Donor Variables	All Patients	Gr 1 0–3 Days	Gr 2 4–7 Days	Gr 3 ≥8 Days	*p*		
	*n* = 241	Total *n* = 92	Total *n* = 80	Total *n* = 69	1 vs. 2	1 vs. 3	2 vs. 3
Age (y)	46 (35–53)	43 (32–51)	46 (33–54)	46 (38–54)	0.35	0.06	0.33
Gender (% male)	54.8	57.6	57.5	47.8	>0.99	0.26	0.25
Height (cm)	175 (168–180)	175 (170–183)	175 (168–180)	173 (166–180)	0.43	0.09	0.35
Weight (kg)	80 (70–85)	80 (70–87)	79 (70–85)	75 (70–81)	0.56	0.28	0.12
Body mass index (kg/m^2^)	25 (23–28)	26 (23–28)	26 (23–29)	25 (23–28)	0.56	0.56	0.21
Left ventricular ejection fraction (%)	60 (55–65)	60 (55–62)	61 (57–68)	61 (55–65)	0.31	0.31	0.32
CPR pre brain death (%)	28.6	29.3	38.8	15.9	0.20	0.06	**0.003**
Donor duration on IMC/ICU (d)	4 (3–8)	2 (2–3)	5 (4–5)	12 (10–16)	**<0.0001**	**<0.0001**	**<0.0001**
Arterial hypertension (%)	49.6	38.9	57.9	57.1	0.09	0.13	>0.99
Diabetes mellitus (%)	16.5	15.9	18.2	16.0	>0.99	>0.99	>0.99
History of smoking (%)	62.7	65.0	65.9	56.1	>0.99	0.37	0.35
History of alcohol abuse (%)	41.8	42.9	49.2	33.3	0.39	0.45	0.09
History of drug abuse (%)	11.8	13.7	10.2	10.9	0.54	0.79	>0.99
Norepinephrine, peak dose (µg/kg/min)	0.12 (0.05–0.26)	0.15 (0.05–0.28)	0.11 (0.04–0.26)	0.12 (0.05–0.24)	0.79	0.16	0.33
Donor cause of death							
ICB (%)	46.1	50.0	28.7	60.9	**0.005**	0.20	**0.0001**
Trauma (%)	22.4	26.1	26.3	13.0	>0.99	**0.05**	0.06
Hypoxic (%)	16.2	15.2	20.0	13.0	0.43	0.82	0.28
Vascular (%)	6.2	3.3	10.0	5.8	0.12	0.70	0.38
Other (%)	9.1	5.4	15.0	7.2	0.12	0.18	>0.99
Laboratory values							
White blood cell count, peak (10^9^/L)	18 (14–23)	17 (13–23)	18 (15–22)	19 (14–23)	0.29	0.70	0.34
C-reactive protein (mg/L)	163 (63–249)	118 (24–218)	185 (103–259)	187 (100–284)	0.40	0.73	0.43
Hemoglobin (g/dL)	10 (8–12)	11 (8–14)	10 (8–12)	9 (8–10)	0.12	**0.00011**	**0.025**
Lactate dehydrogenase (U/L)	323 (238–531)	300 (234–519)	323 (248–509)	333 (242–586)	0.22	0.56	0.36
Sodium (mmol/L)	149 (144–154)	147 (142–151)	153 (145–158)	149 (143–154)	**<0.0001**	0.11	**0.017**
Potassium (mmol/L)	4 (4–5)	4 (4–5)	4 (4–4)	4 (4–4)	0.14	0.64	0.25

**Table 3:** Peri- and postoperative parameters and survival grouped by donor length of stay in the ICU. Median values with interquartile range (Q1–Q3) are shown for continuous variables or percentages for discrete variables. Significance was calculated by Pearson’s chi-square test or, when its application conditions were not met, by Fisher’s exact test for qualitative variables and Student’s *t*-test for quantitative variables.

Outcome and Survival	All Patients	Gr 1 0–3 Days	Gr 2 4–7 Days	Gr 3 ≥8 Days	*p*		
	*n* = 241	Total *n* = 92	Total *n* = 80	Total *n* = 69	1 vs. 2	1 vs. 3	2 vs. 3
Total graft ischemia time (min)	213 (187–237)	214 (193–242)	215 (185–215)	207 (176–207)	0.41	0.24	0.72
Graft cold ischemia time (min)	149 (127–172)	151 (134–176)	147 (119–147)	148 (121–148)	0.39	0.14	0.58
Postoperative hospital stay (d)	36 (27–51)	37 (26–48)	34 (26–34)	39 (28–39)	0.32	0.41	0.08
Postoperative IMC/ICU stay (d)	16 (10–28)	17 (10–30)	15 (10–15)	16 (10–16)	0.21	0.51	0.09
Mechanical ventilation (h)	66 (25–175)	92 (28–201)	54 (26–54)	51 (21–51)	0.30	0.98	0.37
Duration of surgery (min)	412 (339–505)	415 (346–491)	403 (333–403)	411 (341–411)	0.74	0.68	0.49
Blood transfusions surgery							
Packed red blood cells, ml	2970 (1620–4590)	2970 (1620–4455)	2430 (1350–2430)	3375 (1890–3375)	0.69	0.49	0.29
Platelets, ml	880 (660–1540)	880 (660–1540)	880 (660–880)	880 (440–880)	0.33	0.65	0.65
Fresh frozen plasma, ml	1000 (0–2000)	1250 (500–2125)	1000 (0–1000)	1250 (0–1250)	0.20	0.80	0.35
Blood transfusions IMC/ICU							
Packed red blood cells, ml	1890 (810–4320)	1620 (810–4320)	1890 (540–1890)	2160 (810–2160)	0.56	0.62	0.95
Platelets, ml	220 (0–1100)	220 (0–1100)	220 (0–220)	220 (0–220)	0.44	0.18	0.54
Fresh frozen plasma, ml	3500 (2000–6750)	4250 (2250–8000)	3000 (1750–3000)	3500 (2000–3500)	0.76	0.25	0.54
Postoperative morbidity							
Infection/sepsis (%)	23.2	29.7	18.8	19.7	0.11	0.12	>0.99
Rejection within stay (%)	6.8	3.3	11.3	6.1	0.07	0.46	0.38
Hemodialysis post HTx (%)	57.4	60.7	50.6	61.3	0.21	>0.99	0.23
Neurological complications (%)	14.8	13.2	12.5	19.7	>0.99	0.38	0.26
Re-thoracotomy post HTx (%)	30.0	29.7	28.8	31.8	>0.99	0.86	0.72
ECLS post HTx (%)	28.7	31.9	25.0	28.8	0.40	0.73	0.71
Survival							
30-day survival *n* (%)	216/238 (91%)	79/91 (87%)	75/80 (94%)	62/67 (93%)	0.20	0.31	>0.99
1-year survival *n* (%)	161/203 (79%)	61/78 (78%)	57/69 (82%)	43/56 (77%)	0.54	>0.99	0.51

## Data Availability

The data presented in this study are available on request from the corresponding author. The data are not publicly available due to ethical reasons.

## References

[1] Sorabella R.A., Guglielmetti L., Kantor A., Castillero E., Takayama H., Schulze P.C., Mancini D., Naka Y., George I. (2015). Cardiac Donor Risk Factors Predictive of Short-Term Heart Transplant Recipient Mortality: An Analysis of the United Network for Organ Sharing Database. Transplantation Proceedings.

[2] Murana G., Fiorentino M., Gliozzi G., Di Marco L., Potena L., Martin Suarez S., Pacini D., Loforte A. (2020). Donor risk analysis and validation in heart transplants: A single-centre experience. Interact. Cardiovasc. Thorac. Surg..

[3] Smits J.M., De Pauw M., de Vries E., Rahmel A., Meiser B., Laufer G., Zuckermann A. (2012). Donor scoring system for heart transplantation and the impact on patient survival. J. Heart Lung Transplant..

[4] Oehler D., Immohr M.B., Erbel-Khurtsidze S., Aubin H., Bruno R.R., Holst H.T., Westenfeld R., Horn P., Kelm M., Tudorache I. (2022). Intracerebral bleeding in donors is associated with reduced short-term to midterm survival of heart transplant recipients. ESC Heart Fail.

[5] Immohr M.B., Akhyari P., Bottger C., Mehdiani A., Dalyanoglu H., Westenfeld R., Oehler D., Tudorache I., Aubin H., Lichtenberg A. (2021). Cytomegalovirus mismatch after heart transplantation: Impact of antiviral prophylaxis and intravenous hyperimmune globulin. Immun. Inflamm. Dis..

[6] Immohr M.B., Aubin H., Westenfeld R., Erbel-Khurtsidze S., Tudorache I., Akhyari P., Lichtenberg A., Boeken U. (2022). Heart Transplantation of the Elderly-Old Donors for Old Recipients: Can We Still Achieve Acceptable Results?. J. Clin. Med..

[7] Sugimura Y., Immohr M.B., Aubin H., Mehdiani A., Rellecke P., Tudorache I., Lichtenberg A., Boeken U., Akhyari P. (2021). Impact of Reported Donor Ejection Fraction on Outcome after Heart Transplantation. Thorac. Cardiovasc. Surg..

[8] Moitra V.K., Guerra C., Linde-Zwirble W.T., Wunsch H. (2016). Relationship Between ICU Length of Stay and Long-Term Mortality for Elderly ICU Survivors. Crit. Care Med..

[9] Marshall D.C., Hatch R.A., Gerry S., Young J.D., Watkinson P. (2020). Conditional Survival With Increasing Duration of ICU Admission: An Observational Study of Three Intensive Care Databases. Crit. Care Med..

[10] Akbas T. (2019). Long length of stay in the ICU associates with a high erythrocyte transfusion rate in critically ill patients. J. Int. Med. Res..

[11] Mahesh B., Choong C.K., Goldsmith K., Gerrard C., Nashef S.A., Vuylsteke A. (2012). Prolonged stay in intensive care unit is a powerful predictor of adverse outcomes after cardiac operations. Ann. Thorac. Surg..

[12] Nixon J.L., Kfoury A.G., Brunisholz K., Horne B.D., Myrick C., Miller D.V., Budge D., Bader F., Everitt M., Saidi A. (2012). Impact of high-dose inotropic donor support on early myocardial necrosis and outcomes in cardiac transplantation. Clin. Transplant..

[13] Verzelloni Sef A., Sef D., Garcia Saez D., Trkulja V., Walker C., Mitchell J., McGovern I., Stock U. (2021). Heart Transplantation in Adult Congenital Heart Disease with the Organ Care System Use: A 4-Year Single-Center Experience. ASAIO J..

[14] Briceno J., Marchal T., Padillo J., Solorzano G., Pera C. (2002). Influence of marginal donors on liver preservation injury. Transplantation.

[15] Deschenes M., Belle S.H., Krom R.A., Zetterman R.K., Lake J.R. (1998). Early allograft dysfunction after liver transplantation: A definition and predictors of outcome. National Institute of Diabetes and Digestive and Kidney Diseases Liver Transplantation Database. Transplantation.

[16] Cuende N., Miranda B., Canon J.F., Garrido G., Matesanz R. (2005). Donor characteristics associated with liver graft survival. Transplantation.

[17] Markmann J.F., Markmann J.W., Markmann D.A., Bacquerizo A., Singer J., Holt C.D., Gornbein J., Yersiz H., Morrissey M., Lerner S.M. (2001). Preoperative factors associated with outcome and their impact on resource use in 1148 consecutive primary liver transplants. Transplantation.

[18] Hoofnagle J.H., Lombardero M., Zetterman R.K., Lake J., Porayko M., Everhart J., Belle S.H., Detre K.M. (1996). Donor age and outcome of liver transplantation. Hepatology.

[19] Ghobrial R.M., Steadman R., Gornbein J., Lassman C., Holt C.D., Chen P., Farmer D.G., Yersiz H., Danino N., Collisson E. (2001). A 10-year experience of liver transplantation for hepatitis C: Analysis of factors determining outcome in over 500 patients. Ann. Surg..

[20] Cignarella A., Redley B., Bucknall T. (2020). Organ donation within the intensive care unit: A retrospective audit. Aust. Crit. Care.

[21] Hunter A., Johnson L., Coustasse A. (2014). Reduction of intensive care unit length of stay: The case of early mobilization. Health Care Manag..

[22] Angleitner P., Kaider A., Gokler J., Moayedifar R., Osorio-Jaramillo E., Zuckermann A., Laufer G., Aliabadi-Zuckermann A. (2018). High-dose catecholamine donor support and outcomes after heart transplantation. J. Heart Lung Transplant..

[23] Fiorelli A.I., Branco J.N., Dinkhuysen J.J., Oliveira Junior J.L., Pereira T.V., Dinardi L.F., Santos M.M., Dias R.R., Pereira L.A., Stolf N.A. (2012). Risk factor analysis of late survival after heart transplantation according to donor profile: A multi-institutional retrospective study of 512 transplants. Transplantation Proceedings.

[24] D’Aragon F., Lamontagne F., Cook D., Dhanani S., Keenan S., Chasse M., English S., Burns K.E.A., Frenette A.J., Ball I. (2020). Variability in deceased donor care in Canada: A report of the Canada-DONATE cohort study. Can. J. Anaesth.

[25] Cook J., Thomas M. (2017). Cardiac arrest in ICU. J. Intensive Care Soc..

[26] Thongprayoon C., Cheungpasitporn W., Petnak T., Miao J., Qian Q. (2021). Increased short-term and long-term mortality in community- and hospital-acquired hypernatraemia and in patients with delayed serum sodium correction. Int. J. Clin. Pract..

[27] Boeken U., Albert A., Mehdiani A., Petrov G., Westenfeld R., Saeed D., Akhyari P., Lichtenberg A. (2016). Impact of Donor Hypernatremia on Outcome after Cardiac Transplantation. Thorac. Cardiovasc. Surg..

[28] Hoefer D., Ruttmann-Ulmer E., Smits J.M., Devries E., Antretter H., Laufer G. (2010). Donor hypo- and hypernatremia are predictors for increased 1-year mortality after cardiac transplantation. Transpl. Int..

[29] Finger M.A., Cipullo R., Rossi Neto J.M., Dos Santos C.C., Contreras C.A., Chaccur P., Dinkhuysen J.J., de Souza R., Dias Franca J.I., Lin-Wang H.T. (2019). Donor hypernatremia and smoking addiction contribute to primary graft failure in heart transplantation. Clin. Transplant..

[30] Misar A., McLin V.A., Calinescu A.M., Wildhaber B.E. (2022). Impact of length of donor ICU stay on outcome of patients after pediatric liver transplantation with whole and ex situ split liver grafts. Pediatr. Transplant..

[31] Rizvi S.A., Luc J.G.Y., Choi J.H., Phan K., Moncho Escriva E., Patel S., Massey H.T., Tchantchaleishvili V. (2018). Outcomes and survival following heart retransplantation for cardiac allograft failure: A systematic review and meta-analysis. Ann. Cardiothorac. Surg..

[32] Rivinius R., Helmschrott M., Ruhparwar A., Schmack B., Darche F.F., Thomas D., Bruckner T., Doesch A.O., Katus H.A., Ehlermann P. (2020). Elevated pre-transplant pulmonary vascular resistance is associated with early post-transplant atrial fibrillation and mortality. ESC Heart Fail..

